# Non-structural carbohydrates regulated by season and species in the subtropical monsoon broad-leaved evergreen forest of Yunnan Province, China

**DOI:** 10.1038/s41598-018-19271-8

**Published:** 2018-01-18

**Authors:** Wande Liu, Jianrong Su, Shuaifeng Li, Xuedong Lang, Xiaobo Huang

**Affiliations:** 1 Research Institute of Resources Insects, Chinese Academy of Forestry, Kunming, 650224 China; 2 Pu’er Forest Eco-system Research Station, China’s State Forestry Administration, Kunming, 650224 China

## Abstract

Non-structural carbohydrates (NSC) play important roles in adapting to environments in plants. Despite extensive research on the seasonal dynamics and species differences of NSC, the relative contributions of season and species to NSC is not well understood. We measured the concentration of starch, soluble sugar, NSC, and the soluble sugar:starch ratio in leaves, twigs, trunks and roots of twenty dominant species for dry and wet season in monsoon broad-leaved evergreen forest, respectively. The majority of concentration of NSC and starch in the roots, and the leaves contained the highest concentration of soluble sugar. A seasonal variation in starch and NSC concentrations higher in the dry season. Conversely, the wet season samples had higher concentration of soluble sugar and the sugar:starch ratio. Significant differences exist for starch, soluble sugar and NSC concentrations and the sugar:starch ratio across species. Most species had higher starch and NSC concentrations in the dry season and higher soluble sugar concentration and the sugar:starch ratio in wet season. Repeated variance analysis showed that starch and NSC concentrations were strongly affected by season although the effect of seasons, species, and the interaction of the two on the starch, soluble sugar, and NSC concentrations were significant.

## Introduction

Non-structural carbohydrates (NSC) are the primary photosynthates and the key regulators of the physiological adjustment of plants to environmental stress^1–4^. In addition, NSC provide substrates for growth and metabolism^5,6^ and therefore play vital roles in plant’s life processes^7^. The NSC level of a plant is an important indicator of the carbon source and sink capacity of vegetation^8–10^, and can further inform on plant growth^3^, buffering capacity, and adaptation strategies^11^. Hence, quantitative studies of the contribution of NSC to the carbon balance are of crucial importance to understand the survival and growth of plants.

The amounts of non-structural carbohydrates exhibit seasonal trends in order to adapt to seasonal resource variation, such as water, light, temperature, and nutrient availability. Seasonal dynamics of NSC have been studied extensively in most of forest ecosystems. Hoch^12^ reports the highest concentrations in starch, soluble sugar, and NSC in leaves in early summer for deciduous species. In contrast, evergreen conifer trees had hardly any change for soluble sugar in leaves throughout the season. Similarly, the NSC in stem sapwood varied very little throughout the season^12^. While yellow birch and sugar maple seedlings had the highest total NSC and starch by the end of the growing season^13^. In Mediterranean regions, NSC concentration of deciduous *Aesculus californica* dropped during fruit production in fall and re-growth in early spring, whereas evergreen *Quercus agrifolia* showed little change throughout the year^14^. For the tropical forests, Würth *et al*.^2^ reports NSC showed a dry season (dormant period) maximum and a wet season (growing season) minimum in 17 tropical trees. Newell *et al*.^15^ also reports the highest NSC concentrations in dormant period for tropical pioneer species, whereas later succession evergreen species showed smaller or non-significant seasonal changes in NSC concentration. In subtropical regions, Bullock^16^ found the NSC of *Jacaratia mexicana* in stem increased in the end of the rainy season, whereas *Spondias purpurea* showed little change throughout the year. Although seasonal variation in NSCs concentrations is well-documented in most of forest ecosystems, the seasonal trend of NSCs continues to be a subject of active research and some controversy and the relative contributions of seasonal effects to the dynamics of NSC are not well understood.

The dynamics and concentration of NSC display differences across species within the same region that is related to their different life forms or ecological strategies^15,17–20^. The concentrations of NSC and their components differed significantly among species and tissues for three temperate tree species in Northeast China^21^. Similar results were found among other temperate species^12,13,22,23^, Mediterranean species^20^, and tropical species^2^, but the dynamics of NSC also display species specific difference. Several authors have reported differences in the carbohydrate dynamics of deciduous and evergreen species^15^, and most often evergreen species display less dramatic seasonal fluctuations of their carbohydrate reserves than deciduous species^12^. In deciduous plants, NSC is mobilized from the perennial structures to support tissue growth and respiration in early spring, resulting in decreased concentrations of NSC in these storage organs^24^. In woody plants, sugars accumulate from autumn to winter^25^. It has been observed that maximum sugar concentrations are found in poplars in the winter^26,27^. In some herbaceous plants, researchers have also found that changes in sugar concentrations are correlated with season^22^. Therefore, The concentration and dynamics of NSC may be influenced by the species and their taxonomic relationships^28^.

Although NSC seasonal dynamics and species differences in tropical, temperate and Mediterranean forests ecosystems have been studied, the effect of season and species on NSC have not been well characterized in subtropical forests ecosystems. Most evergreen species should display less dramatic seasonal fluctuations in NSC because seasonal variation in carbon gain may be less common for evergreen species in subtropical forests than in temperate forests. In this study, we hypothesize that the NSC in the subtropical monsoon broad-leaved evergreen forest exhibits the greatest seasonal changes due to seasonal precipitation reduction. Precipitation chiefly occurs in the wet season between May and October, and not as frequently during the dry season (from November to April) in Pu’er region of Yunnan Province, China^29^. We also hypothesize that: (1) greatest species fluctuations in NSC concentrations will occur, and (2) the effect of season on the NSC will be higher than species because all species are affected by the season. These hypotheses were tested by seasonally measuring NSC and its component concentrations in leaves, twigs, trunks and roots of twenty of the most abundant species growing in the subtropical monsoon broad-leaved evergreen forest of Yunnan Province, China. The repeated measures analysis of variance was used to distinguish the effect of season and species on NSC, which allows for a more detailed picture of the effect of season and species on NSC.

## Results

### NSC allocation among tissues

**Figure 1:**
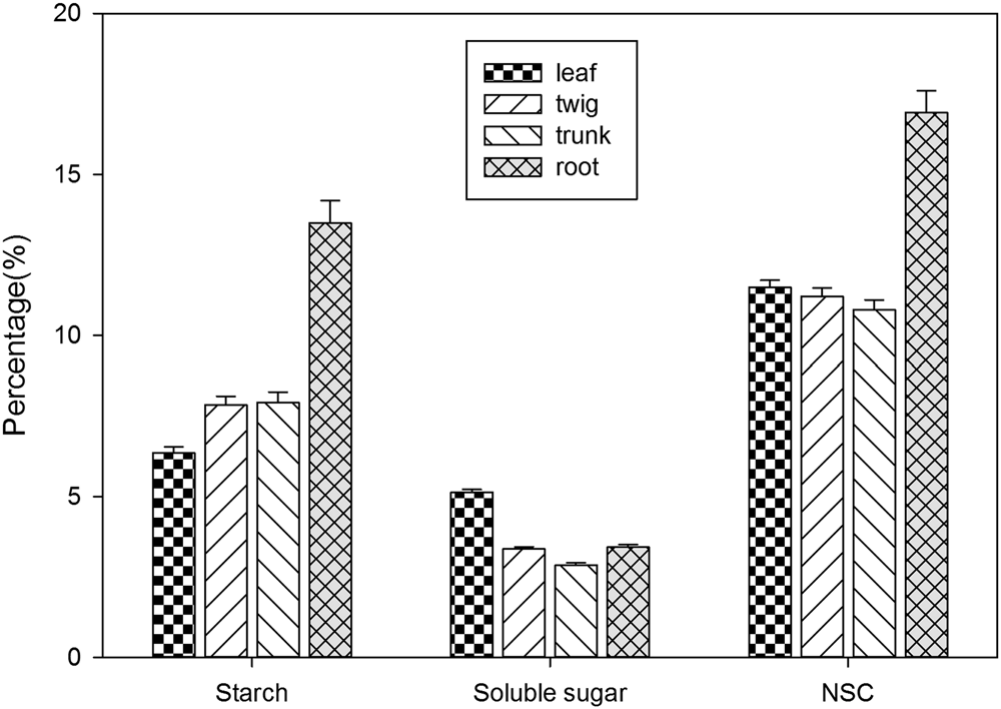
The concentration of starch, soluble sugar, and non-structural carbohydrates for the leaves, twigs, trunks and roots across all seasons.

Starch, soluble sugar and NSC concentrations varied significantly among four tissues across all seasons (Fig. 1). The highest starch and NSC concentrations were found in the roots, while the leaves had the lowest starch concentrations. No significant differences were found for starch concentrations between twigs and trunks and for NSC concentrations among leaves, twigs and trunks (*p* > 0.05). The largest soluble sugar concentration was found to be in the leaves, closely followed by twigs and then roots, and lastly trunks; there were no significant differences between twigs and roots (*p* > 0.05).

### Seasonal differences for NSC

Dramatic variation in the concentration of starch, soluble sugar and NSC and the ratio of soluble sugar to starch occurred between dry and wet seasons (Table 1). The dry season had the higher concentrations of starch and NSC than the wet season. However, the concentration of soluble sugar and the ratio of soluble sugar to starch were higher in the wet season than dry season (Table 1).

**Table 1:** Changes of starch, soluble sugar, non-structural carbohydrates, and the ratio of soluble sugar to starch in different seasons. Different letters in the same row indicate significant differences after Bonferroni’s *post-hoc* test (*p* < 0.05).

Indicators	Seasons	Mean ± SE(%)
Starch	Dry season	13.76 ± 0.33a
	Wet season	3.52 ± 0.07b
Soluble sugar	Dry season	3.28 ± 0.07a
	Wet season	4.16 ± 0.05b
NSC	Dry season	17.05 ± 0.33a
	Wet season	7.69 ± 0.09b
The ratio of soluble sugar to starch	Dry season	0.42 ± 0.03a
	Wet season	1.51 ± 0.03b

**Figure 2:**
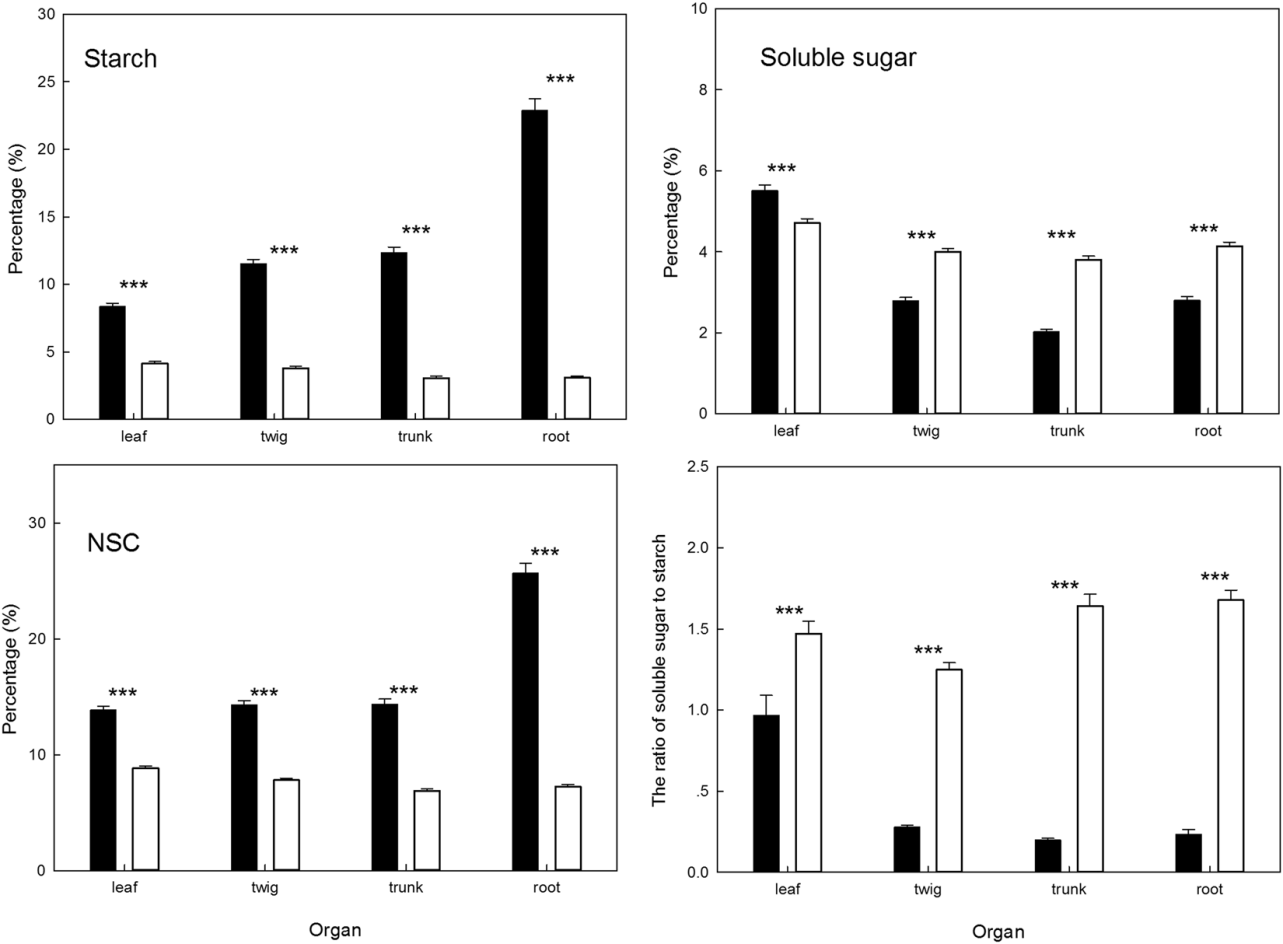
The concentration of starch, soluble sugar, and non-structural carbohydrates and the ratio of soluble sugar to starch for the leaves, twigs, trunks and roots in dry season and wet season, open bars represent the dry season, and the closed bars are the wet season, ** and *** indicate significant difference at the 0.01 level and the 0.001 level, respectively.

Significant seasonal differences were found for the concentration of starch, soluble sugar and NSC and the ratio of soluble sugar to starch in four tissues (leaf, twig, trunk and root) (Fig. 2). The concentration of starch and NSC were higher in the dry season than in wet season for all four tissues. But the concentration of soluble sugar and the ratio of soluble sugar to starch were higher in the wet season than in dry season for all four tissues except for the concentration of soluble sugar in leaf (Fig. 2).

### Species differences for NSC

Significant differences existed among species for the concentration of starch (*F* = 3.709, *p* < 0.000), soluble sugar (*F* = 7.398, *p* < 0.000) and NSC (*F* = 5.125, *p* < 0.000) and the ratio of soluble sugar to starch (*F* = 4.653, *p* < 0.000, Table 2). The concentration of starch, soluble sugar and NSC and the ratio of soluble sugar to starch changes from 5.08% (*Phoebe puwenensis*) to 11.59% (*Tarennoidea wallichii*), 2.86% (*Glochidion lanceolarium*) to 4.84% (*Tarennoidea wallichii*), 8.35% (*Phoebe puwenensis*) to 16.43% (*Tarennoidea wallichii*) and 0.54 (*Phoebe puwenensis*) to 1.49 (*Lithocarpus truncatus*), respectively and the mean are 8.90%, 3.70%, 12.60% and 0.94, respectively.

**Table 2:** The concentrations of starch, soluble sugar, and non-structural carbohydrates and the ratio of soluble sugar to starch for twenty species sampled in this study. 1-*Glochidion lanceolarium*, 2-*Castanopsis calathiformis*, 3-*Anneslea fragrans*, 4-*Castanopsis hystrix*, 5-*Lithocarpus grandifolius*, 6-*Lasianthus chinensis*, 7-*Machilus robusta*, 8-*Gordonia axillaris*, 9-*Castanopsis echidnocarpa*, 10-*Olea rosea*, 11-*Schima wallichii*, 12-*Litsea rubescens*, 13-*Pithecellobium clypearia*, 14-*Lithocarpus fenestratus*, 15-*Lithocarpus truncatus*, 16-*Tarennoidea wallichii*, 17-*Aporusa villosa*, 18-*Rapanea neriifolia*, 19-*Phoebe puwenensis*, 20-*Litsea panamonja*. Different letter indicates significant difference at *p* = 0.05 level.

Species serial number	Starch(%)	Soluble sugar(%)	NSC(%)	The ratio of soluble sugar to starch
1	7.35 ± 1.09abc	2.86 ± 0.24a	10.21 ± 1.07ab	0.71 ± 0.16abc
2	8.38 ± 0.90abc	3.42 ± 0.15ac	11.80 ± 0.89ab	0.79 ± 0.07abc
3	8.63 ± 0.62bc	4.61 ± 0.18be	13.24 ± 0.65ac	1.04 ± 0.07a
4	8.35 ± 0.65 abc	3.46 ± 0.12ac	11.81 ± 0.59ab	0.82 ± 0.06abc
5	8.39 ± 1.74abc	3.12 ± 0.27ad	11.51 ± 1.60ab	1.14 ± 0.24abc
6	9.24 ± 1.16abc	2.94 ± 0.25a	12.18 ± 1.17ab	0.76 ± 0.14abc
7	9.60 ± 0.87bc	3.43 ± 0.16ac	13.04 ± 0.80ac	1.15 ± 0.11a
8	7.79 ± 0.74abc	4.18 ± 0.41ab	11.98 ± 0.85ab	0.69 ± 0.11abc
9	11.55 ± 0.85c	3.31 ± 0.14ac	14.86 ± 00.81a	0.62 ± 0.07bc
10	11.20 ± 1.20bc	4.14 ± 0.19bcde	15.34 ± 1.24a	0.92 ± 0.08abd
11	10.08 ± 1.24abc	4.79 ± 0.16b	14.86 ± 1.25ac	1.04 ± 0.09ab
12	6.99 ± 0.82ab	4.25 ± 0.25bcde	11.25 ± 0.76ab	0.98 ± 0.09abd
13	10.82 ± 2.22abc	3.52 ± 0.29aef	14.34 ± 2.22ab	0.81 ± 0.18abc
14	7.50 ± 0.73ab	3.77 ± 0.14acf	11.27 ± 0.70ab	1.06 ± 0.08a
15	7.49 ± 0.79abc	3.45 ± 0.14ac	10.93 ± 0.75ab	1.49 ± 0.20a
16	11.59 ± 1.56abc	4.84 ± 0.27bf	16.43 ± 1.70ac	0.54 ± 0.05c
17	6.99 ± 0.81ab	3.07 ± 0.16a	10.05 ± 0.78bc	0.84 ± 0.08abc
18	9.76 ± 0.50c	3.39 ± 0.13ac	13.16 ± 0.45ac	0.60 ± 0.05c
19	5.08 ± 0.72a	3.27 ± 0.32aef	8.35 ± 0.73b	1.21 ± 0.21abc
20	9.59 ± 1.98abc	3.40 ± 0.28aef	12.99 ± 1.89ab	0.58 ± 0.07 cd
Mean	8.90 ± 0.22	3.70 ± 0.04	12.60 ± 0.21	0.94 ± 0.03
*F*	3.709	7.398	5.125	4.653
*P*	<0.000	<0.000	<0.000	<0.000

**Figure 3:**
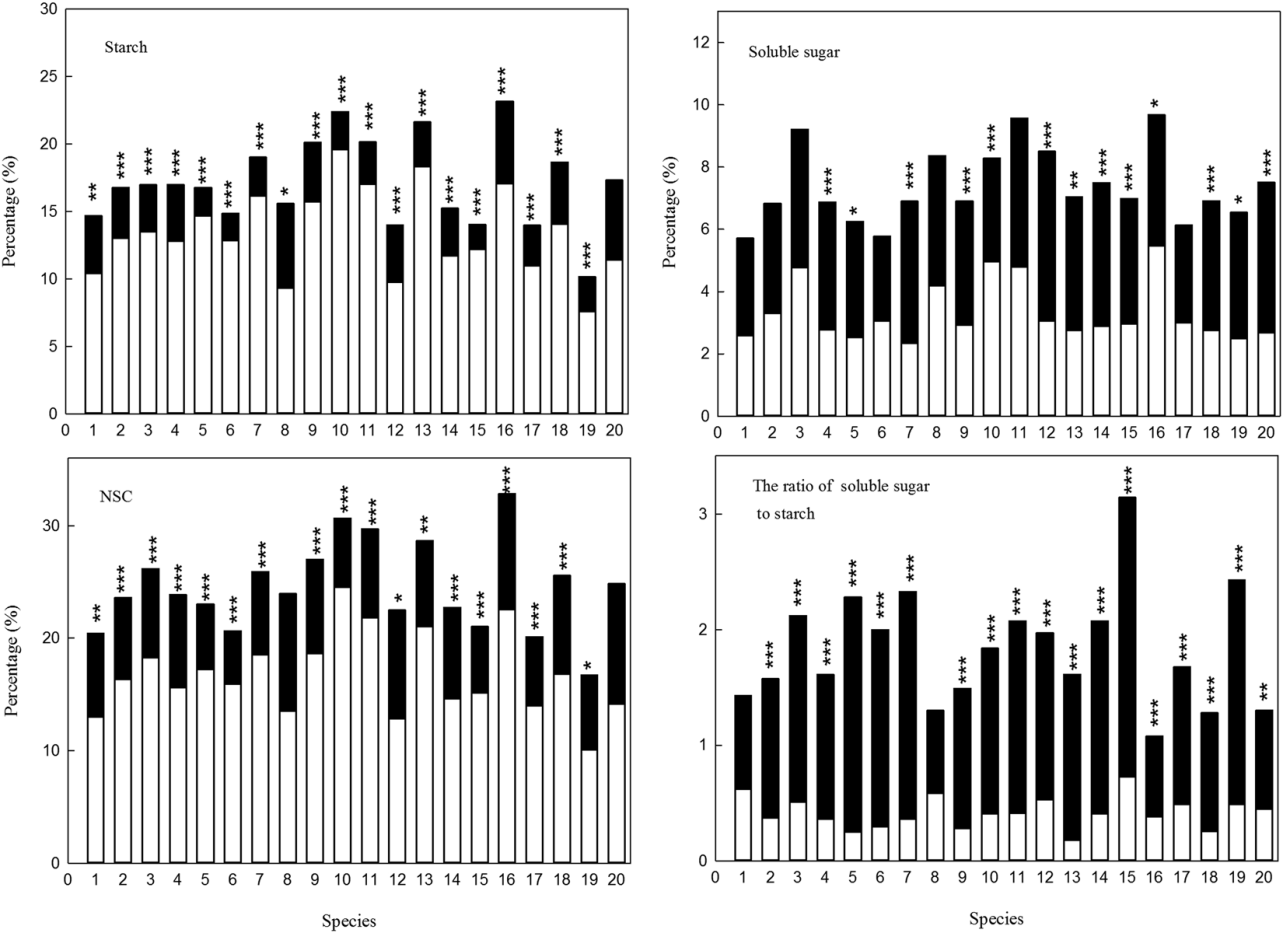
The concentration of starch, soluble sugar, and NSC and the ratio of soluble sugar to starch for twenty species in dry season and wet season, open bars represent the dry season, and the closed bars are the wet season, 1- *Glochidion lanceolarium*, 2-*Castanopsis calathiformis*, 3-*Anneslea fragrans*, 4-*Castanopsis hystrix*, 5-*Lithocarpus grandifolius*, 6-*Lasianthus chinensis*, 7-*Machilus robusta*, 8-*Gordonia axillaris*, 9-*Castanopsis echidnocarpa*, 10-*Olea rosea*, 11-*Schima wallichii*, 12-*Litsea rubescens*, 13-*Pithecellobium clypearia*, 14-*Lithocarpus fenestratus*, 15-*Lithocarpus truncatus*, 16-*Tarennoidea wallichii*, 17-*Aporusa villosa*, 18-*Rapanea neriifolia*, 19-*Phoebe puwenensis*, 20-*Litsea panamonja*. *, ** and *** indicate significant difference at the 0.05 level, 0.01 level and 0.001 level, respectively.

All species except *Litsea panamonja* had higher the concentration of starch in the dry season than in the wet season (Fig. 3). The same trends were found in the concentration of NSC for all species except *Gordonia axillaris* and *Litsea panamonja* (Fig. 3). More than half of species had higher concentrations of soluble sugar in the wet season than in dry season, and only two species (*Olea rosea* and *Tarennoidea wallichii*) had higher concentration of soluble sugar in dry season (Fig. 3). All species except *Glochidion lanceolarium* and *Gordonia axillaris* had a higher ratio of soluble sugar to starch in wet season (Fig. 3).

### Variation sources of non-structural carbohydrates

Species and season effects were significant to the concentrations of starch, soluble sugar and NSC, and a significant interaction was observed between species and season (Table 3). The concentration of starch and NSC were mainly affected by season, while the interaction between species and season was a major factor in the the concentration of soluble sugar (Table 3).

**Table 3:** Summary about the effect of variation from species, season and both interactions on non-structural carbohydrates.

Parameter	Source of variation	SS	*df*	MS	*F*	*P*
Starch	Species	2758.84	19	145.20	3.709	0.000
	Species -error	27401.19	700	39.15		
	Seasons	19944.43	1	19944.43	452.687	0.000
	Species × Season	3729.41	19	196.29	4.455	0.000
	Seasons -error	30840.50	700	44.06		
Soluble sugar	Species	381.97	19	20.10	7.398	0.000
	Species -error	1902.27	700	2.72		
	Seasons	111.96	1	111.96	63.892	0.000
	Species × Season	442.66	19	23.30	13.295	0.000
	Seasons -error	1226.64	700	1.75		
NSC	Species	3743.26	19	197.01	5.125	0.000
	Species -error	26907.55	700	38.44		
	Seasons	17070.35	1	17070.35	396.833	0.000
	Species × Season	5182.12	19	272.74	6.340	0.000
	Seasons -error	30111.50	700	43.02		

## Discussion

### NSC and it components contents

The starch and NSC contents accounted for 13.76% and 17.05% of the dry season-dry weight in this study, respectively. These are higher than temperate species in Northeast China^7,21,30^, *Acer saccharum* and *Betula alleghaniensis* in North America^13^, but within the range of tropical species^2,11,15^. Likewise, The soluble sugar contents in the dry season are also higher than *Betula platyphylla* and *Tilia amurensis*^30^ and *Fraxinus mandschurica*^31^ in China, *Acer saccharum* and *Betula alleghaniensis* in North America^13^. It is also lower than the mean of common species in Northeast China^7^, but still the range of tropical species^2,11^. Unlike the dry season, the NSC contents in the wet season are higher than *Acer saccharum* and *Betula alleghaniensis* in North America^13^, but it remains within temperate species in Northeast China^7,21,30^ and tropical species in Panama^2,11^. The starch contents only accounted for 3.52% of the dry weight in the wet season in this study. That amount is higher than *Quercus mongolica* and *Pinus koraiensis*^21^, *Betula platyphylla* and *Tilia amurensis*^30^ and *Betula alleghaniensis*^13^, but lower than the mean of common species in Northeast China^6^ and *Larix gmelinii*^20^, *Acer saccharum* in North America^13^, and also is within the range of tropical species^2^. As with the dry season, The soluble sugar contents in the wet season are also higher than *Betula platyphylla* and *Tilia amurensis*^30^ and *Fraxinus mandschurica*^31^ in China, *Acer saccharum* and *Betula alleghaniensis* in North America^13^, but lower than the mean of common species in Northeast China^7^, and remains within the range of tropical species^2,11^.

### NSC allocation among tissues

Different allocation patterns of the NSC among tissues may reflect their unique functions. In this study, starch was the main fraction of NSC(>70%) and is the main storage component of carbohydrates. The largest portion of the starch and NSC concentrations was located in the roots across all seasons, as also shown in Gaucher *et al*.^13^, Palacio *et al*.^20^, Loescher *et al*.^32^ and Cruz and Moreno^33^. This indicated that the roots were the main NSC storage tissue in monsoon evergreen broad-leaved forest in the Pu’er region, Yunnan Province, China. Root Carbon storage plays a vital role in maintaining plant physiology when trees suffer aboveground disturbance^34,35^. Species in monsoon evergreen broad-leaved forest have a strong sprouting ability after damage^36^. The high NSC concentrations in the roots may be related to species regeneration strategies because species that respond to damage by resprouting often have high carbohydrate concentrations in their roots^15,37–39^. Conversely, high NSC concentrations in the roots has also been related to species shade-toleraance. Shade-tolerant species usually allocate more NSC to the root for enduring a shady environment^40^, which enhances their survival rate^41,42^, and fuels rapid growth in a suitable environment^40^. In this study, most of studied species are the representative species for monsoon evergreen broad-leaved forests in the Pu’er region and have strong shade-tolerant abilities, which result in the high NSC concentrations in the roots. The lowest starch concentration and the largest soluble sugar concentration in leaves also reflected that the major function for leaves is to produce carbohydrates by photosynthesis and is a NSC source^43^.

### Seasonal dynamics of NSC

Significant seasonal variations were found for all NSC components concentrations in subtropical monsoon broad-leaved evergreen forest in China. The higher starch and NSC concentrations were found in the dry season, and the wet season had the higher soluble sugar concentrations and sugar: starch. Our seasonal NSC variation results are similar to previous studies performed by Bacelar *et al*.^44^, Guehl *et al*.^45^, Li *et al*.^7^, Mooney and Billings^46^, Newell *et al*.^15^, Tissue *et al*.^47^, and Würth *et al*.^2^. The observed differences between the dry and wet season could be due to long-term adaptive responses of species to seasonal environmental conditions^48^ and the seasonal use of NSC for growth^33^. The wet season NSC and starch concentration reduction may reflect the rapid growth of plants. A lot of rain raised the air and soil moisture in the wet season in Pu’er region. Higher moisture and nutrient availability drove plant growth activity. Most of NSC and starches were utilized to support new tissues (such as cambium, new shoots and new fine roots) production in the wet season^15,35^. Meanwhile, plant respiration also consumed some of NSC and starch^12,35^.

Light limitations occur in the wet season due to an increased in cloud cover associated with precipitation^49^ and thus weakened photosynthesis. Furthermore, the higher starch and NSC concentrations in the dry season may be due to translocation of non-structural carbohydrates from senescing leaves and higher rates of photosynthesis at the beginning of the dry season^4,15^. Wang *et al*.^50^ reported that the highest leaf litter occured during February in monsoon evergreen broad-leaved forest in the Pu’er region. These indicated the former month before February, such as January or December, was the period of leaf NSC concentration change for many species in monsoon evergreen broad-leaved forest. Therefore, carbohydrates translocated from senescing leaves and may have contributed to the increase of starch and NSC concentrations. However, higher maximum rates of photosynthesis for some species which have new leaves also added the starch and NSC concentrations^4,15,51^. Evergreen species in seasonally dry environments accumulate carbohydrates during the dry season, because photosynthesis continues while the activities of cambium, xylem and phloem and growth ceases^2,52,53^. Dry seasons enhanced starch and NSC concentrations, indicating that carbon investments were more constrained by the environment than by acquisition methods during these periods^2^.

Although starch has benefits for long-term energy storage in plants^54^, soluble sugars are not only involved in the osmotic adjustment of cells but also serve as signal substances to enable adaptation to environmental changes^7^. The higher soluble sugar concentration in the wet season is not solely due to plants photosynthesis, but also because of the carbohydrates conversion from starch to sugar. However, soluble sugars exhibit more stable seasonal flux in comparison with starch, which is in agreement with the result of Newell *et al*.^15^. As an indicator of environmental change, the proportion of soluble sugars and starch (the sugar:starch ratio) in plants is also regulated by seasonality^55,56^. In our study, the ratio of soluble sugar to starch exhibited large seasonal flux; the sugar:starch ratio in the wet season was about four fold greater than in the dry season. The ratio of soluble sugar to starch was associated with the seasonal fluctuations of starch and soluble sugar concentrations, and the conversion between soluble sugar and starch. The higher soluble sugar concentration, coupled with the lower starch concentration, resulted in the higher ratio of soluble sugar to starch in the wet season. These indicated more carbohydrates were invested in growth during the wet season. Similarly, the lower sugar:starch ratio in the dry season indicated more carbohydrates were invested to long-term energy storage.

### Species specificity of NSC

Although seasonal effects appear to overrule species effects, a high species specificity was found in this study. Significant differences exist among species for the starch, soluble sugar and NSC contents. The concentrations of starch, soluble sugar and NSC of twenty species ranged from 5.08% to 11.59%, 2.86% to 4.84%, 8.35% to 16.43%, respectively. The starch and NSC contents were significantly higher in *Castanopsis echidnocarpa* and *Olea rosea* than in *Phoebe puwenensis*, while *Tarennoidea wallichii* had the highest soluble sugar concentration. Significant species differences have been reported in previous studies^2,7,12,21,30,31,57^ and biological properties play a major role in such differences. Plants living under similar environmental conditions may show different content and allocation of carbohydrates in relation to their life forms or ecological strategies^15,17,20^. Several authors have reported differences in the carbohydrate dynamics of deciduous and evergreen species^7,12,15^. In this study, the higher starch and NSC content was present in *C. echidnocarpa*, *O. rosea*, *Schima wallichii* and *T. wallichii*, but *P. puwenensis* and *Aporusa villosa* had the lower starch and NSC contents. Importantly, *C. echidnocarpa* and *S. wallichii* are the main constructive species and *P. puwenensis* and *A. villosa* are the primary associated species in monsoon evergreen broad-leaved forest in Yunnan Province, China. The difference of starch and NSC contents among these species indicated there were different allocation and use strategies of carbohydrates between the main constructive and associated species. The constructive species maybe have ample carbon gain and storage when they reach the bright-light conditions of the canopy, while carbon gain is low in the forest understory for the associated species. The constructive species can establish in the shade, but need a gap to successfully grow to larger sizes, and often follow a sit-and-wait strategy. The associated species, however, complete their life cycle in the shade and may follow a more conservative strategy.

## Conclusions

Our results suggest that NSC were allocated differently to leaves, twigs, trunks and roots. The highest concentrations of NSC and starch were found to be in the roots and the highest soluble sugar content in the leaves. This result indicated that the roots were the main carbohydrate storage tissue and the leaves was the main carbohydrate producing tissue. The dramatic seasonal and species variations were that the dry season had higher concentrations of starch and NSC. While the the wet season had the higher concentrations of soluble sugar and the soluble sugar:starch ratio across all species and tissues. These results show that season and species affect the starch, soluble sugar and NSC concentrations, although seasonal effects appear to overrule species effects.

## Materials and Methods

### Study site

The study was conducted in a monsoon evergreen broad-leaved forest in the Pu’er region of Yunnan Province in China (22°03′–24°83′N, 99°15′–102°32′E). The elevation ranges from 317 to 3370 meters above sea level, with a mean annual temperature (2000–2010) of 17.7 °C ranging from 13.8 °C in January to 22.8 °C in July and mean annual rainfall (2000–2010) of 1547.6 mm ranging from 14.2 mm in January to 325.6 mm in July. Precipitation chiefly occurs in the wet season between May and October, and not as frequently during the dry season (from November to April). The air humidity ranged from 62.5% in January to 84.2% in July. The sunlight hours was only 113 h in July due to greater cloud cover, but it was up to 252.7 h in January. The soil is a latosol. The soil water contents in the wet season and the dry season are 35.89% and 26.61% respectively. The dominant vegetation type of the study area is monsoon evergreen broad-leaved forest. The dominant tree species in the old-growth forest were *Castanopsis echinocarpa*, *C. hystrix* and *Schima wallichii*, with the understory containing *Ardisia maculosa*, *Fordia microphylla*, *Scleria herbecarpa* and Pteridophyta. Pteridophyta and Orchidaceae grow epiphytically on stems or the forest canopy occurring frequently in all the surveyed forest^29^. In addition, rich buttressed trees and lianas formed an important characteristic of monsoon evergreen broad-leaved forests in the Pu’er region^29^.

### Field sampling

Field sampling was conducted in the dry (December 2013) and wet seasons (July 2014), respectively. Survey data was collected from the 20 most abundant species in six 60 × 60 m plots established in old-growth forest of monsoon evergreen broad-leaved forest (Table 4), which resulted in more than 85% of the total individuals in each plots being sampled. Depending on how many big trees (*DBH* > 22.5 cm), we selected 3 to 18 individuals of each species to sample leaf, twig, trunk and root. *Olea rosea*, *Rapanea neriifolia*, *Gordonia axillaris*, *Litsea panamonja* and *Aporusa villosa* species did not have individuals with *DBH* greater than 22.5 cm. For those species, we selected individuals with *DBH* greater than 10 cm to sample leaf, twig, trunk and root.

**Table 4:** The information of species sampled in experiments.

Species serial number	Species	Family	Genus
1	*Glochidion lanceolarium*	Euphorbiaceae	*Glochidion*
2	*Castanopsis calathiformis*	Fagaceae	*Castanopsis*
3	*Anneslea fragrans*	Theaceae	*Anneslea*
4	*Castanopsis hystrix*	Fagaceae	*Castanopsis*
5	*Lithocarpus grandifolius*	Fagaceae	*Lithocarpus*
6	*Lasianthus chinensis*	Rubiaceae	*Lasianthus*
7	*Machilus robusta*	Lauraceae	*Machilus*
8	*Gordonia axillaris*	Theaceae	*Gordonia*
9	*Castanopsis echidnocarpa*	Fagaceae	*Castanopsis*
10	*Olea rosea*	Oleaceae	*Olea*
11	*Schima wallichii*	Theaceae	*Schima*
12	*Litsea rubescens*	Lauraceae	*Litsea*
13	*Pithecellobium clypearia*	Leguminosae	*Pithecellobium*
14	*Lithocarpus fenestratus*	Fagaceae	*Lithocarpus*
15	*Lithocarpus truncatus*	Fagaceae	*Lithocarpus*
16	*Tarennoidea wallichii*	Rubiaceae	*Tarennoidea*
17	*Aporusa villosa*	Euphorbiaceae	*Aporusa*
18	*Rapanea neriifolia*	Myrsinaceae	*Rapanea*
19	*Phoebe puwenensis*	Lauraceae	*Phoebe*
20	*Litsea panamonja*	Lauraceae	*Litsea*

For each individual, we sampled two twigs from the outer layer, inner layer and middle layer of the crown, respectively, using a pole tree pruner or artificial climbing trees. All leaves and twigs from the same individual were pooled respectively as two mixed samples for carbohydrate analysis. An increment borer (5 mm diameter) was used to extract a core of trunk tissue. Samples were taken from approximately 1 m height above the ground. Each individual was excavated up to 30–40 cm from its rooting point and only medium sized roots (2–5 cm diameter) were selected as root samples. After sampling, leaf, twig, trunk and root samples were immediately stored in a sealed bag and taken back to the laboratory and dried at 80 °C to a constant weight as soon as possible.

### Carbohydrate analyses

Dried leaves, twigs, trunk and roots were sieved separately using a metal-free plastic mill to pass through a 0.4 mm-pore mesh screen into particles for carbohydrates analysis. Soluble sugars were extracted from 0.05 g ground material in 10 ml 80% (v/v) ethanol. The extraction was done in a shaking water bath at 80 °C. After centrifugation, the concentration of soluble sugars was determined colorimetrically at 490 nm using the modified phenol–sulphuric method^16^. Starch contained in the pellet remaining after the extraction of soluble sugars was hydrolyzed to glucose. After incubating in sodium acetate and amyloglucosidase solution, the concentration of starch was determined colorimetrically at 490 nm using the modified phenol–sulphuric method as detailed by Newell *et al*.^16^. The sum of soluble sugars and starch are referred to as total non-structural carbohydrates (NSC).

### Statistical analyses

We present species, tissue and season specific data. The contents of soluble sugars, starch, NSC, and the ratio of soluble sugar to starch were expressed as a concentration (%). The soluble sugars, starch, NSC contents, and the sugar to starch ratio for different tissues were expressed as the mean with treating all individual sample as replications. The soluble sugars, starch, NSC contents, and the sugar to starch ratio data from individual samples of one season were pooled for each tissue sample dates within seasons. All tissue sample dates were pooled into seasonal dates. The one-way analysis of variance (ANOVA) was used to determine the tissue differences of the concentrations of starch, soluble sugar, and NSC across all seasons. The same ANOVA technique was used to assess the seasonal differences of starch, soluble sugar, NSC and the sugar:starch ratio for the same tissue. The seasonal differences of starch, soluble sugar, NSC and the sugar:starch ratio were also obtained through ANOVA. Within species, the data on soluble sugars, starch, and NSC contents and the sugar:starch ratio for individual trees were expressed as the mean of four tissues, and all statistical tests treat individual trees as replicates. Similarly, the species differences in the concentrations of starch, soluble sugar, and NSC and the ratio of soluble sugar to starch among all species was tested using ANOVA. Before performing the ANOVA, the data were tested for a normal distribution and variance homogeneity, and when necessary data were log transformed. Effects of species, seasons, and the interaction of species and season interactions on the concentrations of starch, soluble sugar, and NSC were tested by repeated measures analysis of variance. Statistical analyses were conducted using SPSS19.0 (SPSS Inc., Chicago, USA), with significant differences defined at p values less than 0.05.
